# Long-Term Infection and Vertical Transmission of a Gammaretrovirus in a Foreign Host Species

**DOI:** 10.1371/journal.pone.0029682

**Published:** 2012-01-03

**Authors:** Toshie Sakuma, Jason M. Tonne, Jessica A. Malcolm, Tayaramma Thatava, Seiga Ohmine, Kah-Whye Peng, Yasuhiro Ikeda

**Affiliations:** Department of Molecular Medicine, Mayo Clinic, Rochester, Minnesota, United States of America; University of Oxford, United Kingdom

## Abstract

Increasing evidence has indicated natural transspecies transmission of gammaretroviruses; however, viral-host interactions after initial xeno-exposure remain poorly understood. Potential association of xenotropic murine leukemia virus-related virus (XMRV) in patients with prostate cancer and chronic fatigue syndrome has attracted broad interests in this topic. Although recent studies have indicated that XMRV is unlikely a human pathogen, further understanding of XMRV xenoinfection would allow *in vivo* modeling of the initial steps of gammaretroviral interspecies transmission, evolution and dissemination in a new host population. In this study, we monitored the long-term consequences of XMRV infection and its possible vertical transmission in a permissive foreign host, wild-derived *Mus pahari* mice. One year post-infection, XMRV-infected mice showed no notable pathological changes, while proviral DNA was detected in three out of eight mice. XMRV-infected mice remained seropositive throughout the study although the levels of gp70 Env- and p30 capsid-specific antibodies gradually decreased. When vertical XMRV transmission was assessed, no viremia, humoral immune responses nor endogenization were observed in nine offspring from infected mothers, yet one offspring was found PCR-positive for XMRV-specific sequences. Amplified viral sequences from the offspring showed several mutations, including one amino acid deletion in the receptor binding domain of Env SU. Our results therefore demonstrate long-term asymptomatic infection, low incidence of vertical transmission and limited evolution of XMRV upon transspecies infection of a permissive new host, *Mus pahari*.

## Introduction

Gammaretroviruses usually transmit within a single host species [1], [2], often from mother to offspring [3], [4]. However, studies have also identified a number of probable natural interspecies transmissions between different species of placental mammals, placental and marsupial mammals, and mammals to birds [5]–[7]. For instance, recent transspecies transmission and ongoing endogenization of a gammaretrovirus have been implicated in koala retrovirus (KoRV), which causes lymphoid neoplasias in captive and wild koalas [8]–[10]. Since KoRV is closely related to gibbon ape leukemia virus and woolly monkey virus [11]–[13], these three viruses are considered to be from a common ancestor [8], [12], likely a close relative of an endogenous gammaretrovirus from an Asian wild mouse [6]. However, the initial steps of gammaretroviral interspecies transmission, adaptation and dissemination in a new host population remains poorly understood.

Previously, due to the possible concerns over the potential zoonotic transmission of endogenous retroviruses, the xenotropic infection by porcine endogenous retroviruses (PERV) has been extensively studied in immunocompromised mice or patients transplanted with pig organs. Those studies found the lack of evidence for PERV transmission in nonhuman primate models or humans transplanted or treated with porcine tissues [14]–[16], while modest PERV replication was observed in mice transgenic for a human PERV receptor [17]. The experimental transspecies transmission of KoRV was also assessed in rats. KoRV was able to establish a productive infection in all rats at an early stage (3 weeks post infection), while the virus was efficiently cleared in most animals by 8 weeks. Of note, one KoRV-infected rat developed a fibrosarcoma with high levels of KoRV proviral DNA [5]. Those observations demonstrate rather efficient viral clearance upon artificial xenoinfection of gammaretroviruses.

Murine gammaretroviruses, including exogenous and endogenous murine leukemia viruses (MLVs), were obtained from inbred mice and feral mice trapped in the wild, and these viruses were classified into various categories based on the host range and receptor usage [18]–[21]. Ecotropic MLVs infect only murine cells, while amphotropic, polytropic and xenotropic MLVs can infect non-murine cells. Identification of xenotropic murine leukemia virus-related virus (XMRV) and MLV-like sequences in patients with prostate cancer [22] and chronic fatigue syndrome [23], [24] has attracted further interests in the transspecies transmission of MLV and MLV-related viruses. Although recent studies have casted the doubt over the authenticity of XMRV and MLV-like viruses as human pathogens [25]–[30], understanding of XMRV infection in a foreign host could provide unique insights into how a gammaretrovirus replicates, adapts and disseminates in a new host species. We have previously reported early events in XMRV infection of an Asian wild mouse species with functional Xpr1 receptor for XMRV entry [31], [32], [33]. XMRV proviral DNA was detected in spleen, blood, and brain at five to twelve weeks post-intraperitoneal infection [33]. Mice infected with XMRV mounted adaptive immune responses against XMRV as evidenced by the production of neutralizing antibody for XMRV Env and Gag [33]. Prominent G-to-A hypermutations were also found in viral genomes isolated from the spleen, suggesting intracellular restriction of XMRV infection by APOBEC3 *in vivo* [33]. XMRV is also restricted by both *Fv1^n^* and *Fv1^b^* [34]. However *Mus pahari* lacks the *Fv1* restriction gene because *Fv1* was acquired in the *Mus* lineage after *Mus pahari* diverged [35]. These data support the use of XMRV and *Mus pahari* as a model for transspecies transmission of a gammaretrovirus.

In this study, we examined the long-term consequences of XMRV infection and possible vertical transmission of XMRV from mother to offspring. Mice infected with XMRV remained seropositive over one year. At one year post-infection, no notable pathological changes were observed, while proviral DNA was detected in three out of eight infected mice. When nine offspring from XMRV-infected parents were examined, only one offspring was found positive for XMRV proviral DNA. Our results therefore demonstrate the long-term control and low incidence of vertical transmission of XMRV upon transspecies infection of a permissive foreign host, *Mus pahari*.

## Results and Discussion

**Figure 1:**
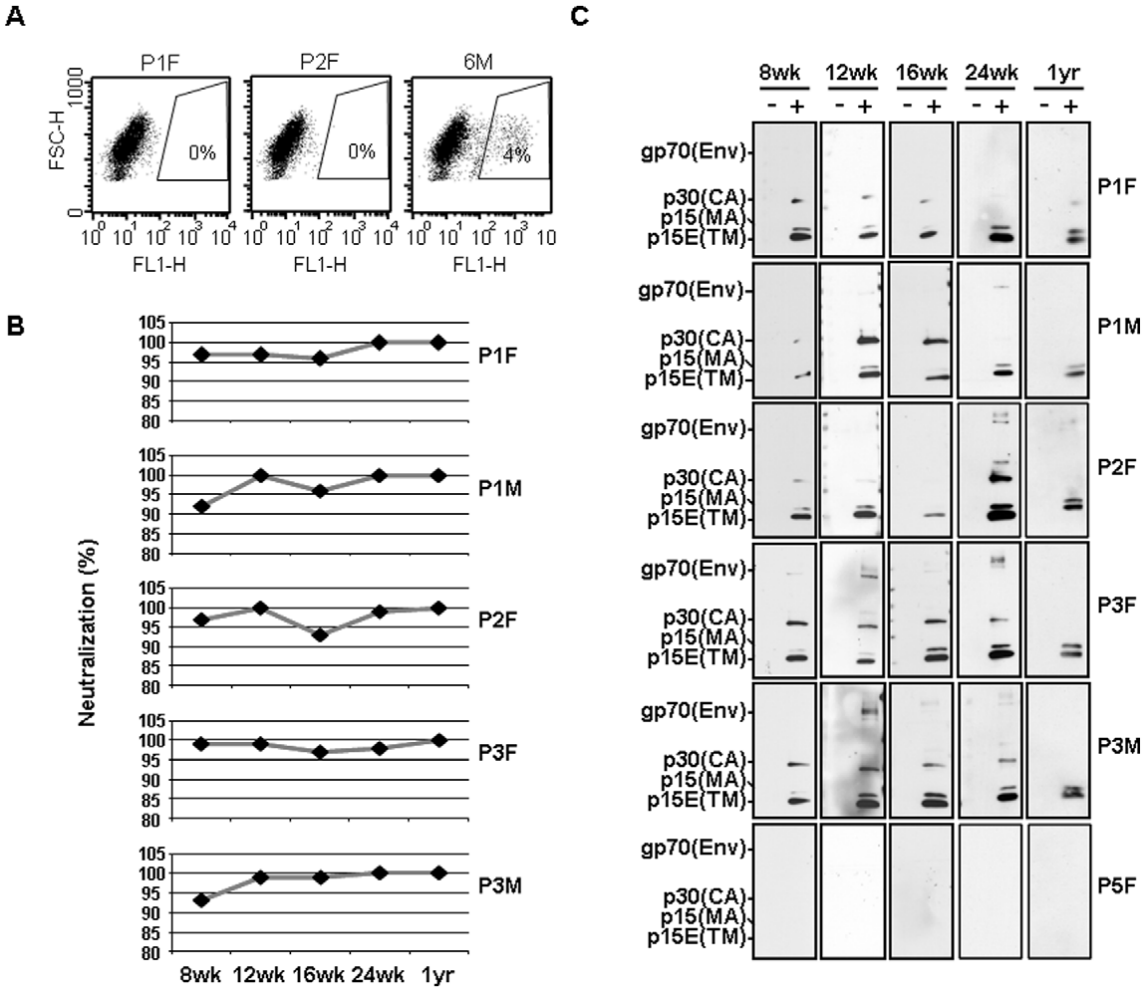
Sustained anti-XMRV humoral immunity in mice chronically infected with XMRV. (A) Neutralization assay was carried out using GFP-carrying XMRV as described previously [33], [52]. Flow cytometry analysis of virus neutralization activities of plasma samples from XMRV-infected mice (P1F and P2F) is compared to those from a control mouse (6M). (B) Results from neutralization assay are summarized in percentage which was calculated as the reciprocal of infectivity with a maximum infectivity being determined by incubation of the virus with an uninfected mouse serum. Data from Parent 1 female (P1F), P1 male (P1M), P2F, P3F, and P3M are shown. (C) XMRV proteins were detected by Western blotting with plasma samples of XMRV-infected mice. Results from five (P1F, P1M, P2F, P3F, and P3M) mice and a control (p5F) are shown. Viral proteins, envelope, Env (gp70), capsid, CA (p30), envelope transmembrane, TM (p15E), and matrix, MA (p15) are indicated. XMRV antigen negative and positive are indicated as − and +, respectively. Blood samples were collected at 8 wk, 12 wk, 16 wk, 24 wk and 1 year p.i.

We have previously reported on the early events of XMRV infection in *Mus pahari* [33]. Using these wild-derived mice, which have no endogenous MLV-like sequences in its genome and lack the potent retroviral restricting Fv1 activity [36]–[38], we examined the long-term consequences of the virus infection. A total of 8 mice which showed XMRV seroconversion were used in this study [29], [33]. Plasma samples of these infected mice showed strong XMRV-neutralizing activity (i.e., 96±0.011, 99±0.004, 95±0.012, 98±0.009, and 100±0 percent neutralization, at 12 wk, 16 wk, 24 wk and 1 year p.i., respectively, ± standard error) at a dilution of 1∶20 (Fig. 1A and B). Specificity of these neutralizing antibodies was further verified by Western blotting (Fig. 1C); plasma from these mice with high neutralizing activities could detect XMRV-specific structural proteins, i.e., Env glycoprotein (gp70), capsid protein (CA; p30), matrix protein (MA; p15) and transmembrane envelope protein (TM; p15E), over one year. In general, plasma from infected mice at 12–24 wk p.i. readily detected all the virus proteins (Fig. 1C), while most plasma from mice at 1 year p.i. still showed immune reactivity against Env gp70 and CA p30.

We also examined the biodistribution of XMRV in mice chronically infected with XMRV. Although we previously detected XMRV-specific sequences in blood, heart, brain, prostate, and testis tissues at 2 and 4 wk p.i. [33], XMRV DNA was not detected from blood or plasma in any mice after 8 wk p.i.. No viral RNA was detected from plasma samples at any time point tested. We were also unable to isolate XMRV from infected mice at 8 wk p.i. through co-cultivation of blood cells with 293T cells, followed by 10 passages. These results suggested the lack of active viral production after the early stage of viral infection, most likely due to the sustained XMRV-specific humoral immune response.

In acute XMRV infection, some mice showed increased numbers of white blood cells (WBC) including lymphocytes, monocytes, or granulocytes [33]. We therefore monitored the blood parameters of XMRV-infected mice over one year (Tables S1, S2, S3, S4, S5). The 95% reference range was calculated as (mean −1.96× SD) to (mean +1.96× SD) using CBC data from 12 uninfected *Mus pahari* as determined previously [33] and used as indicative values. Of note, one control mouse (6M) had mild thrombocytosis at early stage of the study (Table S1). The numbers of WBC and red blood cells (RBC) mostly fit within the normal range of blood parameters for *Mus pahari* (normal ranges for WBC and RBC between 4.4∼8.6×10^9^/L and 9.1∼12.1×10^12^/L, respectively) [33]. Although transient reduction in numbers of WBC were observed in two animals at 24 wk p.i. (0.94 and 2.96×10^9^/L; Table S4) with abnormally low lymphocyte numbers (i.e., 0.78 and 2.5×10^9^/L, respectively), these numbers were returned to normal at 1 year p.i. (Table S5). The lymphocytosis found in most treated mice cannot be accurately linked to XMRV infection due to the lack of appropriate age-matched controls. (Table S4). RBC counts including hemoglobin (HGB) and mean corpuscular hemoglobin concentration (MCHC) were generally within normal range at each time point analyzed except for 24 wk p.i. (Table S4), and the number of RBCs went back to normal at 1 year p.i. (Table S5). In addition, marginal reductions in MCHC were observed in 6 out of 9 mice at 1 year p.i. (Table S5). When we analyzed the blood chemistry at 1 year p.i., concentrations of albumin, alkaline phosphatase, alanine transferase, amylase, blood urea nitrogen, calcium (not ionized), creatinine, globulin, glucose, potassium, sodium, phosphorus, total bilirubin, and total protein were all within normal ranges, indicating that long-term XMRV infection did not affect the metabolic and hepatic functions, and renal function did not show a significant pathology.

To analyze the possible pathological abnormalities and the presence of XMRV proviral DNA, we sacrificed the mice at 1 year p.i.. Five XMRV infected mice plus one control mouse showed notable calcification in pancreas, a sign commonly seen in chronic pancreatitis, while no notable pathological changes was observed in the other mice. We also tested the presence of XMRV provirus in the organs including heart, brain, spleen, lung, kidney, pancreas, bladder, testis, prostate, and lymph node by real-time qPCR as reported previously [29]. Most samples were negative for XMRV-specific DNA, including the pancreas with notable calcification. From the tissues analyzed, three samples were qPCR-positive (the pancreases from animal P1F and P4F had 150 and 190 genome copies/µg, respectively, and the spleen in mouse P2F had 30 genome copies/µg). We amplified and sequenced the *env* region of XMRV by nested PCR to compare to a sequence used to infect the mice. Nine mutations, from T to C (×4), G to T (×2), G to C (×1), A to T (×1) and T to G (×1) in a total of 4959 bp (from nucleotide (nt) position 5864 to nt 6414, based on GenBank accession no. NC_007815) sequenced, and three mutations, from C to T (×2) and A to G (×1) in a total of 4408 bp sequenced, were found in the pancreas-derived DNA from mice P1F and P4F, respectively. Five mutations, from G to A (×2), T to C (×1), G to T (×1), and C to T (×1) in a total of 5510 bp (from nt 5864 to nt 6414 analyzed based on GenBank accession no. NC_007815) sequenced were found in the spleen of mouse P2F. These results demonstrated relatively low levels of genetic mutations in XMRV *env* gene upon long-term infection, which is likely due to poor viral replication in this mouse model.

**Figure 2:**
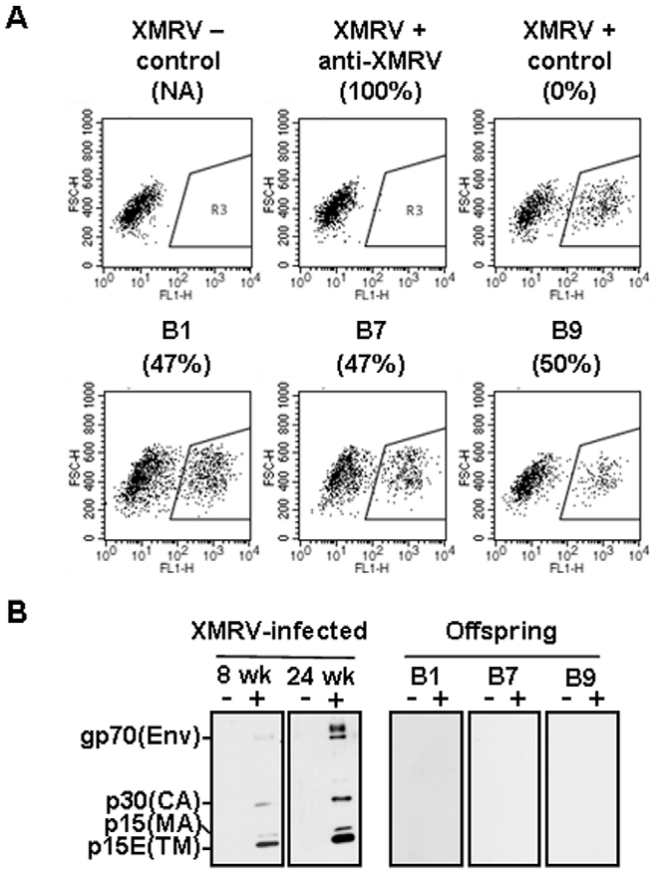
Detection of anti-XMRV antibodies in the offspring from XMRV-infected mice. (A) Flow cytometry data from 293T cells is indicated as XMRV negative (−) control. XMRV-GFP virus was pre-treated with plasma samples from infected mice (XMRV positive (+) anti-XMRV), non-infected mice (XMRV positive (+) control), and offspring (B1, B7, and B9) at a dilution of 1∶20, and then used to infect 293T cells. Numbers shown in parentheses are the percentages of neutralization, which was calculated as the reciprocal of infectivity with a maximum infectivity being determined by incubation of the virus with an uninfected mouse serum. NA, not applicable. (B) XMRV proteins were detected by Western blotting. Data from mice infected with XMRV after 8 and 24 weeks p.i. as positive controls and three offspring (B1, B7, and B9) are shown. Sizes of expected viral proteins are indicated to the left side of the Western blotting: envelope glycoprotein, Env (gp70), capsid, CA (p30), transmembrane, TM (p15E), and matrix, MA (p15).

It is well documented that exogenous MLV strains, which share high sequence similarities with XMRV, are vertically transmitted from mother to offspring [3], [39]–[42]. In order to examine the possible vertical transmission of XMRV, offspring that were born from XMRV-infected mice were included in this study. Three breeding pairs of *Mus pahari* mice (Parents: P1 to P3) that produced offspring were included in this study. From three breeding pairs, a total of nine offspring (Offspring B1 to B3 are from P1, offspring B4 to B7 are from P2, and offspring B8 and B9 are from P3) were produced. Using these mice, we first examined if the plasma from offspring neutralize XMRV. Although plasma samples from XMRV-infected mothers could completely block XMRV infection (∼100% neutralization, Fig. 1A), none of the offspring showed strong XMRV neutralizing activities (Fig. 2A). Plasma from these offspring also failed to detect XMRV structural proteins by Western blotting (Fig. 2B). These results indicate that offspring did not receive the maternal immunity against XMRV nor develop anti-XMRV antibodies.

**Figure 3:**
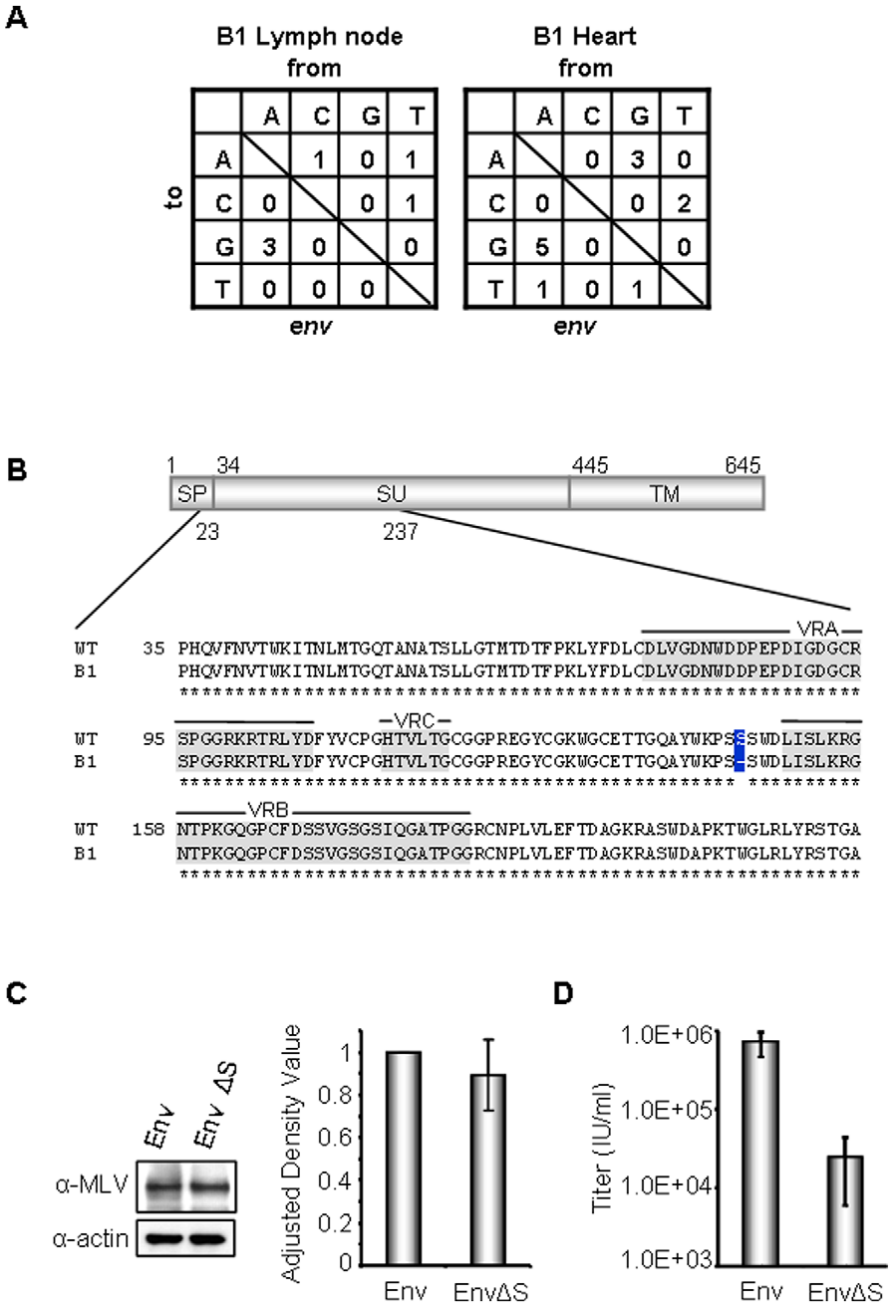
Sequence analysis of *env* gene from XMRV proviral DNA-positive offspring samples. (A) Nested-PCR, cloning, and sequence analysis were performed as described previously [33]. The number of mutations in the *env* gene from lymph node and heart of the mouse B1 are summarized. A total of 2751 bp and 4366 bp were sequenced from lymph node and heart, respectively. (B) Schematic representation of XMRV Env with partial amino acid sequence of receptor binding domain (RBD) is shown. One Env isolate from heart (B1: GenBank accession no. JN987227) has one amino acid deletion of serine at position 148 in SU as boxed in blue. Amino acids from 1 to 33, from 34 to 444, and from 445 to 645 indicate signal peptide (SP), surface protein (SU), and transmembrane protein (TM), respectively. Variable regions (VRA, VRB, and VRC) are highlighted in gray. (C) Protein expression of wild-type XMRV Env and XMRV Env with S148 mutation was analyzed by Western blotting. Env protein was visualized with goat anti-MLV antibody (1∶2000, ATCC). Actin (1∶2000, sigma) was also detected as a loading control. Statistical analysis showed that protein expression was not statistically significant (*p* = 0.31). (D) Retroviral vectors pseudotyped with XMRV Env or XMRV Env with S148 mutation were produced by transient transfection in 293T cells. The transduction titer of retrovirus pseudotyped with XMRV Env or XMRV Env with a serine mutation is statistically significant (*p* = 0.009). Error bars indicate 95% confidence intervals.

The lack of anti-XMRV antibodies in the offspring could be due to no vertical XMRV transmission or due to immunological tolerance to XMRV antigens in infected offspring. Our attempts to isolate XMRV from the offspring, through co-cultivation of offspring blood cells with 293T cells, followed by serial passages for four weeks, were unsuccessful. We then analyzed the presence of XMRV-specific proviral DNA in the tissue samples including brain, heart, lung, liver, spleen, kidney, lymph node, bladder, and prostate by real-time qPCR as described previously [29], [33]. XMRV-positive samples were only found from one offspring, B1 (0.3 and 24 copies of viral genome sequences/µg in tissues of lymph node and heart DNA samples, respectively). Partial *env* gene was amplified from these tissues, cloned and sequenced, and the variations in the recovered viral sequences were determined. We obtained fourteen PCR clones and *env* gene from nt 5864 to nt 6414 bp (NC_007815) was sequenced. All the identified sequences were closely related to XMRV, rather than exogenous MLV CasE #1, which was previously detected in *Mus pahari* [33] or endogenous MLV-like sequences in laboratory mice. Although sequencing analysis found no notable G-to-A mutations in the *env* gene (Fig. 3A), one sequence from B1 (GenBank accession no. JN987227) showed a deletion of three nucleotides (Fig. 3B) resulting in a single amino acid (S148) deletion in the receptor binding domain of the Env SU region. SU glycoprotein variable regions VRA, VRB, and VRC (Fig. 3 B) are known to determine receptor specificity [22], [43]. However, the deletion mutation fell in a region not known to influence receptor interactions.

The S148 deletion was found within the receptor binding domain of XMRV Env (Fig. 3C). To address the influence of this mutation on viral infectivity, we introduced the same mutation into a retroviral vector system, and examined the effects on retroviral infectivity. We first verified the expression of the S148-deleted XMRV Env by Western blotting (Fig. 3C). When retrovirus vectors with wildtype XMRV Env or the mutated Env were produced as described previously [44], the vector with the S148 deletion showed an approximately 10-fold lower titer than that of wild-type vector (Fig. 3D). This observation demonstrated that the S148 mutation in the SU region impairs viral infectivity. We speculate that immune escape from XMRV-specific antibodies through the S148 deletion mutation impaired the viral fitness. Alternatively, this mutation was the consequence of viral adaptation to *Mus pahari* Xpr1 receptor, rather than human XPR1. Further study using cell lines expressing Xpr1 variants will address these questions.

Studies of exogenous MLVs in laboratory mice have demonstrated frequent mother-to-offspring transmission, particularly through milk [39], [45]. For instance, a study using a temperature-sensitive Moloney murine leukemia virus has demonstrated that about 39% of transmission occurs *in utero* (up to day 20 of gestation) and nearly 100% transmission occurs if the offspring suckle breast milk from the infected mothers [46]. On the other hand, reduced vertical transmission is often observed when MLV-infected mice have circulating antibodies [39], [47], [48]. Our study showed inefficient mother-to-offspring XMRV transmission. Offspring were born from XMRV-infected mothers that had high levels of circulating anti-XMRV antibodies at the time of delivery. Thus, low incidence of vertical transmission is likely due to the lack of chronic viremia in *Mus pahari* infected with XMRV. Our attempts to detect XMRV-specific sequences in the milk from XMRV-infected mother mice were unsuccessful, due to technical difficulties to harvest milk from lactating mice.

*In utero* infection or neonatal infection of exogenous MLVs often results in immunological tolerance, leading to frequent viremia in newborn mice [3], [39]–[42]. It is notable that 78% of offspring were viremic in Lake Casitas wild mice that were naturally infected with an exogenous MLV [41]. In contrast, MLV infection at a late postnatal stage often results in induction of passive immunity and asymptomatic infection [49]. In our study, we were unable to isolate XMRV from blood, or to detect viral RNA in the circulation (data not shown), indicating no sustained viremic offspring from XMRV-infected mothers. One offspring was viral DNA positive, but showed no viremia. Thus, we speculate that this mouse was infected by XMRV at a late postnatal stage, most likely through breast milk. The lack of detectable antibodies against XMRV in this offspring might be due to recent XMRV exposure, or limited XMRV replication in this mouse.

In summary, we demonstrate that XMRV shows a very limited replication in a permissive host over a period of one year. We also showed the possible vertical XMRV transmission from mother to offspring with a low frequency. No endogenization was observed in the nine offspring born from XMRV-positive mothers. Recently, poor replication of XMRV was also reported in XMRV-infected rhesus monkeys [50]. This may also explain why transspecies gammaretroviral transmission does not occur frequently; as it is likely a difficult task for a gammaretrovirus to overcome both potent retroviral restriction factors, such as APOBEC3s [33], [51], and sustained adaptive immunity in a new host species.

## Materials and Methods

### Mice

The wild-derived mice, *Mus pahari*/*EiJ*, were purchased from the Jackson Laboratory. The mice were housed in the Mayo Clinic Animal Facility under the Association for Assessment and Accreditation of Laboratory Animal Care (AALAC) guidelines with animal use protocols approved by the Mayo Clinic Animal Use and Care Committee (IACUC number: A24509). All animal experiments were carried out according to the provisions of the Animal Welfare Act, PHS Animal Welfare Policy, the principles of the NIH Guide for the Care and Use of Laboratory Animals, and the policies and procedures of Mayo Clinic. A total of 10 mice received approximately 2.2×10^8^ genomic copies (3.0×10^5^ infectious units of XMRV/mouse) of XMRV intraperitoneally at 8 to 12 weeks of age. XMRV infection was confirmed by the seroconversion as described previously [29], [33]. For vertical transmission analysis, offspring born from XMRV-infected parents were weaned at 4 weeks old and euthanized at 8 weeks old.

### Hematology analysis and blood chemistry

Blood samples were collected at 8 wk, 12 wk, 16 wk, 24 wk, and 1 year p.i. and analyzed by VetScanHM2 Hematology System (Abaxis). At 1 year p.i., blood chemistry was evaluated by Comprehensive Diagnostic Profile (Abaxis).

### Neutralization assay

A GFP vector pseudotyped with XMRV was used for neutralization assay as previously reported (24). Plasma samples were heat inactivated at 56°C for 30 min. The samples at a dilution of 1∶20 were incubated with 2.5×10^4^ infectious units (IU) of GFP-carrying XMRV at 37°C for 30 min. 5×10^4^ 293T cells (ATCC) were infected for 3 days and GFP-positive cell populations were analyzed by flow cytometry (FACscan). GFP-positive cell populations were gated by excluding XMRV negative control cell population. Boxed regions show GFP-positive cells with percentages. Viral titers were calculated as infective units per milliliter (IU/ml) as described previously [44].

### qPCR

DNA from mice tissues and blood samples were extracted by using a PureLink genomic DNA mini kit (Invitrogen) according to the manufacturer's protocol. For the real-time PCR assay, TaqMan Universal PCR Master Mix (Roche), probes (Roche) and primers were used as described previously using ABI 7300 real-time PCR [29], [33].

### Detection of XMRV DNA in blood and tissue samples and viral RNA in plasma samples

DNA from mouse blood and tissue was extracted by using PureLink genomic DNA mini kit (Invitrogen) and viral RNA from plasma samples was extracted by QIAamp viral RNA mini kit (QIAGEN). cDNA from viral RNA was synthesized by RNA to cDNA EcoDry Premix (Clontech) and qPCR was performed to detect XMRV sequences. qPCR positive samples were further analyzed by nested PCR with platinum Taq polymerase (Invitrogen). XMRV *gag* was PCR amplified using the outer primers 5′-ACGAGTTCGTATTCCCGGCCGCA-3′ and 5′-CCGCCTCTTCTTCATTGTTC-3′ and the inner primers 5′-GCCCATTCTGTATCAGTTAA-3′ and 5′-AGAGGGTAAGGGCAGGGTAA-3′. For the XMRV *env* amplification, the outer primers 5′- ATGGAAAGTCCAGCGTTCTCAAA-3′ and 5′- ATGGGGACGCGGGGCCCTACATTG-3′ and the inner primers 5′- AGGAGCCTCAGTACAACGTGACAG-3′ and 5′- TGGCGGGTCAGAGAGAACAGGG-3′ were used. The PCR products were then cloned into TOPO vector (Invitrogen), and the amplified sequences were analyzed by DNADynamo (BlueTractor Software).

### Western blotting

The virus antigen was prepared by transfecting 293T cells with pcDNA3.1 (−)/VP62. Three days p.i., supernatants were collected, filtered through 45 µm filter and centrifuged through 20% sucrose cushion at 13,000 rpm at 4°C. They were centrifuged at the same conditions with PBS. The virus particles were then resuspended in Laemmli sample buffer containing β-mercaptoethanol, and boiled at 95°C for 5 min. Protein samples (10 µl) were subjected for SDS-PAGE with a 4–15% gradient gel (Bio-Rad), and transferred to a polyvinylidene difluoride membrane. They were stained with plasma samples at dilution of 1∶100, followed by ant-mouse IgG antibody (1∶2000). Protein expression was quantified by Image-J and the adjusted density value was calculated by the following link: http://lukemiller.org/index.php/2010/11/analyzing-gels-and-western-blots-with-image-j/. For statistical analysis, Student's *t*-test was performed.

### Production of Retrovirus vectors

Retrovirus vector was created by transfecting a MLV packaging plasmid (0.25 µg), a green fluorescent protein (GFP) vector plasmid (0.5 µg), and an Env-encoding plasmid (0.25 µg) using FuGENE-6 (Roche). Three days after transfection, supernatants were filtered through 0.45-µm filters (Millipore) and 5×10^4^ of 293T cells were transduced with the vectors in the presence of polybrene (8 mg/ml). At three days post-transduction, cells were fixed with 4% paraformaldehyde, and GFP-positive cells were determined by flow cytometry (FACScan). Titers were calculated as infective units per milliliter (IU/ml) as described previously [44].

## Supporting Information

Table S1 **CBC test results at 8 week post-infection.**
(DOC)

Table S2 **CBC test results at 12 week post-infection.**
(DOC)

Table S3 **CBC test results at 16 week post-infection.**
(DOC)

Table S4 **CBC test results at 24 week post-infection.**
(DOC)

Table S5 **CBC test results at 1 year post-infection.**
(DOC)
