# Supplementary material for: Long-Term Infection and Vertical Transmission of a Gammaretrovirus in a Foreign Host Species
Source: PLoS One. 2012 Jan 3;7(1):e29682. doi: 10.1371/journal.pone.0029682 (PMC3250474; doi:10.1371/journal.pone.0029682)
Supplement: Table S1 — CBC test results at 8 week post-infection. (DOC) [file pone.0029682.s001.doc]

**Supplemental Table 1.** CBC test results at 8 week post-infection*^a^*.

| Parameter*^b^* | P1F | P1M | P2F | P2M | P3F | P3M | P4F | P4M | 5M | 6M | **Nomal range***^c^* |
| --- | --- | --- | --- | --- | --- | --- | --- | --- | --- | --- | --- |
| WBC | 7.69 | 3.9 | 7.53 | 3.8 | 7.09 | 6.59 | 6.67 | 6.98 | 6.78 | 7.38 | **4.4-8.6** |
| LYM | 5.91 | 3.4 | 6.04 | 3.27 | 5.21 | 4.85 | 5.98 | 5.53 | 4.58 | 5.43 | **3.4-5.9** |
| MON | 0.05 | 0.07 | 0.39 | 0.04 | 0.22 | 0.26 | 0.11 | 0.29 | 0.04 | 0.52 | **0.01-0.32** |
| GRA | 1.73 | 0.43 | 1.1 | 0.49 | 1.65 | 1.48 | 0.57 | 1.17 | 2.15 | 1.43 | **0.4-2.9** |
| RBC | 11.26 | 10.57 | 11.61 | 7.85 | 11.41 | 10.93 | 11.25 | 11.65 | 11.56 | 10.46 | **9.1-12.1** |
| HGB | 17.7 | 16.1 | 17.7 | 12.2 | 17.8 | 17.5 | 16.7 | 18.7 | 18.8 | 16.7 | **14.3-19.2** |
| HCT | 48.69 | 41.19 | 46.17 | 33.68 | 48.79 | 47.09 | 44.73 | 49.13 | 49.35 | 43.04 | **38-52** |
| MCV | 43 | 39 | 40 | 43 | 43 | 43 | 40 | 42 | 43 | 41 | **40-45** |
| MCH | 15.7 | 15.2 | 15.2 | 15.5 | 15.6 | 16 | 14.9 | 16.1 | 16.3 | 16 | **14.8-16.8** |
| MCHC | 36.4 | 39 | 38.2 | 36.2 | 36.5 | 37.1 | 37.4 | 38.1 | 38.1 | 38.9 | **35.8-38.7** |
| PLT | 552 | 261 | 745 | 33*^d^* | 777 | 874 | 409 | 568 | 175 | 1147 | **244-1042** |

*^a^* Numbers above the normal range of control mice are boxed. Numbers below the normal range of control mice are highlighted.

*^b^* WBC, LYM, MON, GRA, RBC, HGB, HCT, MCV, MCH, MCHC, and PLT represent the white blood cell count (10^9^/L), lymphocyte count (10^9^/L), monocyte count (10^9^/L), granulocyte count (10^9^/L), red blood cell count (10^12^/L), hemoglobin level (g/dL), hematocrit (%), mean corpuscular volume (fL), mean corpuscular hemoglobin (pg), mean corpuscular hemoglobin concentration (g/dL), and platelet count (10^9^/L), respectively.

*^c^* The 95% reference range was calculated as (mean – 1.96 × SD) to (mean + 1.96 × SD) using CBC data from 12 uninfected *Mus pahari* as determined previously (Sakuma et al., 2011) and is shown as the normal range. SD, standard deviation.

*^d^* The hematology profile of P2M may suggest marrow-suppression or most likely blood-clots in the tube. The data is based on the automatic cell counters (VetScanHM2 Hematology System from Abaxis). PLT was not counted on a slide. The mice did not suffer from bleeding after the biopsy.
