# Supplementary material for: Long-Term Infection and Vertical Transmission of a Gammaretrovirus in a Foreign Host Species
Source: PLoS One. 2012 Jan 3;7(1):e29682. doi: 10.1371/journal.pone.0029682 (PMC3250474; doi:10.1371/journal.pone.0029682)
Supplement: Table S2 — CBC test results at 12 week post-infection. (DOC) [file pone.0029682.s002.doc]

**Supplemental Table 2.** CBC test results at 12 week post-infection*^a^*.

| Parameter*^b^* | P1F | P1M | P2F | P2M | P3F | P3M | P4F | P4M | 5M | 6M | **Nomal range***^c^* |
| --- | --- | --- | --- | --- | --- | --- | --- | --- | --- | --- | --- |
| WBC | 5.75 | 7.67 | 7.96 | NA*^d^* | 5.68 | NA | 4.61 | 6.42 | 8.66 | 2.78 | **4.4-8.6** |
| LYM | 4.18 | 6.27 | 5.83 | NA | 4.28 | NA | 2.6 | 4.68 | 5.57 | 2 | **3.4-5.9** |
| MON | 0.3 | 0.36 | 0.29 | NA | 0.34 | NA | 0.39 | 0.36 | 0.05 | 0.21 | **0.01-0.32** |
| GRA | 1.26 | 1.03 | 1.83 | NA | 1.05 | NA | 1.62 | 1.38 | 3.03 | 0.56 | **0.4-2.9** |
| RBC | 10.3 | 9.57 | 10 | NA | 9.6 | NA | 10 | 9.99 | 8.94 | 8.99 | **9.1-12.1** |
| HGB | 16.2 | 14.5 | 14.7 | NA | 15.7 | NA | 14.9 | 15.7 | 14.5 | 14.4 | **14.3-19.2** |
| HCT | 45.5 | 39.1 | 39.4 | NA | 42.5 | NA | 40.3 | 42.7 | 39.2 | 38.6 | **38-52** |
| MCV | 44 | 41 | 39 | NA | 44 | NA | 40 | 43 | 44 | 43 | **40-45** |
| MCH | 15.7 | 15.1 | 14.6 | NA | 16.4 | NA | 14.9 | 15.8 | 16.2 | 16 | **14.8-16.8** |
| MCHC | 35.6 | 36.9 | 37.3 | NA | 36.9 | NA | 37 | 36.8 | 37 | 37.3 | **35.8-38.7** |
| PLT | 469 | 552 | 343 | NA | 632 | NA | 467 | 685 | 950 | 913 | **244-1042** |

*^a^* Numbers above the normal range of control mice are boxed. Numbers below the normal range of control mice are highlighted.

*^b^* WBC, LYM, MON, GRA, RBC, HGB, HCT, MCV, MCH, MCHC, and PLT represent the white blood cell count (10^9^/L), lymphocyte count (10^9^/L), monocyte count (10^9^/L), granulocyte count (10^9^/L), red blood cell count (10^12^/L), hemoglobin level (g/dL), hematocrit (%), mean corpuscular volume (fL), mean corpuscular hemoglobin (pg), mean corpuscular hemoglobin concentration (g/dL), and platelet count (10^9^/L), respectively.

*^c^* The 95% reference range was calculated as (mean – 1.96 × SD) to (mean + 1.96 × SD) using CBC data from 12 uninfected *Mus pahari* as determined previously (Sakuma et al., 2011) and is shown as the normal range. SD, standard deviation.

*^d^* Not available.
