# Supplementary material for: Long-Term Infection and Vertical Transmission of a Gammaretrovirus in a Foreign Host Species
Source: PLoS One. 2012 Jan 3;7(1):e29682. doi: 10.1371/journal.pone.0029682 (PMC3250474; doi:10.1371/journal.pone.0029682)
Supplement: Table S3 — CBC test results at 16 week post-infection. (DOC) [file pone.0029682.s003.doc]

**Supplemental Table 3.** CBC test results at 16 week post-infection*^a^*.

| Parameter*^b^* | P1F | P1M | P2F | P2M | P3F | P3M | P4F | P4M | 5M | 6M | **Nomal range***^c^* |
| --- | --- | --- | --- | --- | --- | --- | --- | --- | --- | --- | --- |
| WBC | 6.98 | 6.42 | 8.24 | 7.2 | 6.11 | 5.74 | 8.01 | NA*^d^* | 6.4 | 5.99 | **4.4-8.6** |
| LYM | 5.4 | 5.32 | 6.18 | 5.52 | 4.7 | 4.28 | 6.45 | NA | 4.53 | 4.52 | **3.4-5.9** |
| MON | 0.38 | 0.05 | 0.47 | 0.13 | 0.63 | 0.13 | 0.24 | NA | 0.16 | 0.28 | **0.01-0.32** |
| GRA | 1.2 | 1.04 | 1.59 | 1.55 | 0.78 | 1.33 | 1.32 | NA | 1.7 | 1.19 | **0.4-2.9** |
| RBC | 9.98 | 9.1 | 9.76 | 9.52 | 10.19 | 9.64 | 9.93 | NA | 9.74 | 9.07 | **9.1-12.1** |
| HGB | 15.9 | 14.1 | 15 | 15.9 | 16.7 | 16.6 | 16.1 | NA | 16.1 | 14 | **14.3-19.2** |
| HCT | 44.48 | 38.07 | 40.62 | 41.55 | 44.36 | 44.68 | 40.37 | NA | 45.06 | 37.64 | **38-52** |
| MCV | 45 | 42 | 42 | 44 | 44 | 46 | 41 | NA | 46 | 41 | **40-45** |
| MCH | 15.9 | 15.5 | 15.4 | 16.7 | 16.4 | 17.2 | 16.2 | NA | 16.5 | 15.4 | **14.8-16.8** |
| MCHC | 35.7 | 37.2 | 36.9 | 38.2 | 37.6 | 37.1 | 39.9 | NA | 35.7 | 37.2 | **35.8-38.7** |
| PLT | 505 | 761 | 564 | 534 | 656 | 853 | 756 | NA | 772 | 908 | **244-1042** |

*^a^* Numbers above the normal range of control mice are boxed. Numbers below the normal range of control mice are highlighted.

*^b^* WBC, LYM, MON, GRA, RBC, HGB, HCT, MCV, MCH, MCHC, and PLT represent the white blood cell count (10^9^/L), lymphocyte count (10^9^/L), monocyte count (10^9^/L), granulocyte count (10^9^/L), red blood cell count (10^12^/L), hemoglobin level (g/dL), hematocrit (%), mean corpuscular volume (fL), mean corpuscular hemoglobin (pg), mean corpuscular hemoglobin concentration (g/dL), and platelet count (10^9^/L), respectively.

*^c^* The 95% reference range was calculated as (mean – 1.96 × SD) to (mean + 1.96 × SD) using CBC data from 12 uninfected *Mus pahari* as determined previously (Sakuma et al., 2011) and is shown as the normal range. SD, standard deviation.

*^d^* Not available.
