# Supplementary material for: Long-Term Infection and Vertical Transmission of a Gammaretrovirus in a Foreign Host Species
Source: PLoS One. 2012 Jan 3;7(1):e29682. doi: 10.1371/journal.pone.0029682 (PMC3250474; doi:10.1371/journal.pone.0029682)
Supplement: Table S4 — CBC test results at 24 week post-infection. (DOC) [file pone.0029682.s004.doc]

**Supplemental Table 4.** CBC test results at 24 week post-infection*^a^*.

| Parameter*^b^* | P1F | P1M | P2F | P2M | P3F | P3M | P4F | P4M | 5M | 6M | **Nomal range***^c^* |
| --- | --- | --- | --- | --- | --- | --- | --- | --- | --- | --- | --- |
| WBC | 6.04 | 7.19 | 8.85 | 6.94 | 6.47 | 2.96 | 8.75 | NA*^d^* | 0.94 | 8.96 | **4.4-8.6** |
| LYM | 4.4 | 6.16 | 6.61 | 5.23 | 4.81 | 2.5 | 7.31 | NA | 0.78 | 6.75 | **3.4-5.9** |
| MON | 0.42 | 0.1 | 0.45 | 0.48 | 0.33 | 0.06 | 0.08 | NA | 0.01 | 0.26 | **0.01-0.32** |
| GRA | 1.22 | 0.93 | 1.79 | 1.23 | 1.33 | 0.4 | 1.37 | NA | 0.15 | 1.96 | **0.4-2.9** |
| RBC | 10.83 | 9.75 | 10.62 | 10.4 | 9.06 | 6.79 | 10.72 | NA | 2.59 | 9.33 | **9.1-12.1** |
| HGB | 16.5 | 14.5 | 15.7 | 15.8 | 14.4 | 10.2 | 15.2 | NA | 3.6 | 13.9 | **14.3-19.2** |
| HCT | 46.91 | 39.86 | 43.06 | 45 | 39.97 | 30.49 | 43.18 | NA | 11.37 | 39 | **38-52** |
| MCV | 43 | 41 | 41 | 43 | 44 | 45 | 40 | NA | 44 | 42 | **40-45** |
| MCH | 15.2 | 14.8 | 14.8 | 15.2 | 15.9 | 15 | 14.2 | NA | 13.8 | 14.9 | **14.8-16.8** |
| MCHC | 35.1 | 36.3 | 36.6 | 35.1 | 36.1 | 33.4 | 35.2 | NA | 31.3 | 35.7 | **35.8-38.7** |
| PLT | 457 | 344 | 649 | 523 | 276 | 343 | 560 | NA | 52 *^e^* | 619 | **244-1042** |

*^a^* Numbers above the normal range of control mice are boxed. Numbers below the normal range of control mice are highlighted.

*^b^* WBC, LYM, MON, GRA, RBC, HGB, HCT, MCV, MCH, MCHC, and PLT represent the white blood cell count (10^9^/L), lymphocyte count (10^9^/L), monocyte count (10^9^/L), granulocyte count (10^9^/L), red blood cell count (10^12^/L), hemoglobin level (g/dL), hematocrit (%), mean corpuscular volume (fL), mean corpuscular hemoglobin (pg), mean corpuscular hemoglobin concentration (g/dL), and platelet count (10^9^/L), respectively.

*^c^* The 95% reference range was calculated as (mean – 1.96 × SD) to (mean + 1.96 × SD) using CBC data from 12 uninfected *Mus pahari* as determined previously (Sakuma et al., 2011) and is shown as the normal range. SD, standard deviation.

*^d^* Not available.

*^e^* The hematology profile of 5M may suggest marrow-suppression or most likely blood-clots in the tube. The data is based on the automatic cell counters (VetScanHM2 Hematology System from Abaxis). PLT was not counted on a slide. The mice did not suffer from bleeding after the biopsy.
