# Supplementary material for: Long-Term Infection and Vertical Transmission of a Gammaretrovirus in a Foreign Host Species
Source: PLoS One. 2012 Jan 3;7(1):e29682. doi: 10.1371/journal.pone.0029682 (PMC3250474; doi:10.1371/journal.pone.0029682)
Supplement: Table S5 — CBC test results at 1 year post-infection. (DOC) [file pone.0029682.s005.doc]

**Supplemental Table 5.** CBC test results at 1 year post-infection*^a^*.

| Parameter*^b^* | P1F | P1M | P2F | P2M | P3F | P3M | P4F | P4M | 5M | 6M | **Nomal range***^c^* |
| --- | --- | --- | --- | --- | --- | --- | --- | --- | --- | --- | --- |
| WBC | 7.23 | 6.58 | 8.34 | 7.01 | 6.91 | 11.15 | 7.51 | NA*^d^* | 8.04 | 6.97 | **4.4-8.6** |
| LYM | 5.17 | 4.37 | 6.7 | 4.89 | 5.07 | 8.06 | 6.13 | NA | 6 | 5.81 | **3.4-5.9** |
| MON | 0.41 | 0.54 | 0.21 | 0.35 | 0.52 | 0.51 | 0.21 | NA | 0.05 | 0.04 | **0.01-0.32** |
| GRA | 1.64 | 1.67 | 1.43 | 1.76 | 1.32 | 2.58 | 1.17 | NA | 1.99 | 1.13 | **0.4-2.9** |
| RBC | 9.17 | 9.63 | 10.67 | 10.04 | 10.44 | 10.74 | 11.07 | NA | 10.17 | 10.31 | **9.1-12.1** |
| HGB | 13.2 | 14.2 | 15.7 | 15.4 | 16.4 | 15.8 | 15.7 | NA | 15.8 | 15.2 | **14.3-19.2** |
| HCT | 38.08 | 41.73 | 42.94 | 42.58 | 46.03 | 45.84 | 44.41 | NA | 44.47 | 41.99 | **38-52** |
| MCV | 42 | 43 | 40 | 42 | 44 | 43 | 40 | NA | 44 | 41 | **40-45** |
| MCH | 14.4 | 14.7 | 14.7 | 15.4 | 15.7 | 14.7 | 14.2 | NA | 15.6 | 14.7 | **14.8-16.8** |
| MCHC | 34.6 | 34 | 36.5 | 36.2 | 35.7 | 34.5 | 35.4 | NA | 35.6 | 36.2 | **35.8-38.7** |
| PLT | 617 | 410 | 628 | 423 | 734 | 545 | 387 | NA | 586 | 607 | **244-1042** |

*^a^* Numbers above the normal range of control mice are boxed. Numbers below the normal range of control mice are highlighted.

*^b^* WBC, LYM, MON, GRA, RBC, HGB, HCT, MCV, MCH, MCHC, and PLT represent the white blood cell count (10^9^/L), lymphocyte count (10^9^/L), monocyte count (10^9^/L), granulocyte count (10^9^/L), red blood cell count (10^12^/L), hemoglobin level (g/dL), hematocrit (%), mean corpuscular volume (fL), mean corpuscular hemoglobin (pg), mean corpuscular hemoglobin concentration (g/dL), and platelet count (10^9^/L), respectively.

*^c^* The 95% reference range was calculated as (mean – 1.96 × SD) to (mean + 1.96 × SD) using CBC data from 12 uninfected *Mus pahari* as setermined previously (Sakuma et al., 2011) and is shown as the normal range. SD, standard deviation.

*^d^* Not available.
